# An in vitro methodology for discovering broadly-neutralizing monoclonal antibodies

**DOI:** 10.1038/s41598-020-67654-7

**Published:** 2020-07-01

**Authors:** Shirin Ahmadi, Manuela B. Pucca, Jonas A. Jürgensen, Rahel Janke, Line Ledsgaard, Erwin M. Schoof, Christoffer V. Sørensen, Figen Çalışkan, Andreas H. Laustsen

**Affiliations:** 1 Department of Biotechnology and Biosafety, Graduate School of Natural and Applied Sciences, Eskisehir Osmangazi University, Eskisehir, Turkey; 2 Department of Biotechnology and Biomedicine, Technical University of Denmark, Kongens Lyngby, Denmark; 3 Medical School, Federal University of Roraima, Boa Vista, Roraima Brazil; 4 Department of Biology, Faculty of Science and Letters, Eskisehir Osmangazi University, Eskisehir, Turkey

**Keywords:** Biotechnology, Drug discovery

## Abstract

Broadly-neutralizing monoclonal antibodies are of high therapeutic utility against infectious diseases caused by bacteria and viruses, as well as different types of intoxications. Snakebite envenoming is one such debilitating pathology, which is currently treated with polyclonal antibodies derived from immunized animals. For the development of novel envenoming therapies based on monoclonal antibodies with improved therapeutic benefits, new discovery approaches for broadly-neutralizing antibodies are needed. Here, we present a methodology based on phage display technology and a cross-panning strategy that enables the selection of cross-reactive monoclonal antibodies that can broadly neutralize toxins from different snake species. This simple in vitro methodology is immediately useful for the development of broadly-neutralizing (polyvalent) recombinant antivenoms with broad species coverage, but may also find application in the development of broadly-neutralizing antibodies against bacterial, viral, and parasitic agents that are known for evading therapy via resistance mechanisms and antigen variation.

## Introduction

Snakebite envenoming causes 80,000–130,000 deaths and around three times as many amputations and other permanent disabilities each year^1^. For the time being, antivenoms containing animal-derived polyclonal antibodies are the only effective therapy against this debilitating condition. Nevertheless, many of these medicines have limited neutralization capacity against the venoms that they are indicated for. This is in part due to the low immunogenicity of some of the most medically important snake toxin families^2,3^, which may fail to raise a strong antibody response in the production animal. As an alternative, the use of well-defined mixtures of recombinant human monoclonal antibodies (recombinant antivenoms) has been demonstrated to hold therapeutic promise^4^. However, for such products to be feasible and cost-competitive to manufacture, the number of monoclonal antibodies included in the formulation of a recombinant antivenom must be limited^5^. As snake venoms are highly complex and comprise multitudes of different toxins from different protein families, this puts a strong requirement on the monoclonal antibodies to be broadly-neutralizing^6^. Therefore, rational approaches for designing broadly-neutralizing monoclonal antibodies that may cover entire subfamilies of toxins are gravely needed.

African cobra snakes are notorious in sub-Saharan Africa for causing death and destitution to a large number of victims each year, as their potent venoms may cause paralysis and severe local tissue damage upon injection into human victims^7–9^. In particular, spitting cobras such as *Naja nigricollis* and *N. mossambica*, are among the snakes whose bites lead to a high number of amputations or permanent disfigurements on the African continent^8^, due to the high abundance of cytotoxins (CTxs) in their venoms^9^. CTxs belong to the three-finger toxin family (3FTx) and interact with and disrupt cellular membranes. This leads to progressive and painful swelling of the bite wound with blistering and bruising that may evolve into tissue necrosis and gangrene^3,10^. As CTxs are of significant medical importance in human envenomings, poorly neutralized by existing antivenoms due to their low immunogenicity, and abundantly available in a multitude of different isoforms of varying similarity, these toxins constitute relevant targets for broadly-neutralizing antibodies. Phospholipases A_2_ (PLA_2_s) also contribute to the necrotic effects caused by spitting cobra venoms^3^, however, it has previously been demonstrated for *N. nigricollis* venom that inhibition of PLA_2_s using *p*-bromophenacyl bromide only modifies the appearance of necrotic regions, but does not reduce the necrotic area in mice^11^. For this reason, and in pursuit of developing a systematic methodology for discovering broadly-neutralizing antibodies against snake toxins, we chose to focus on CTxs obtained from African cobras as model antigens.

**Figure 1:**
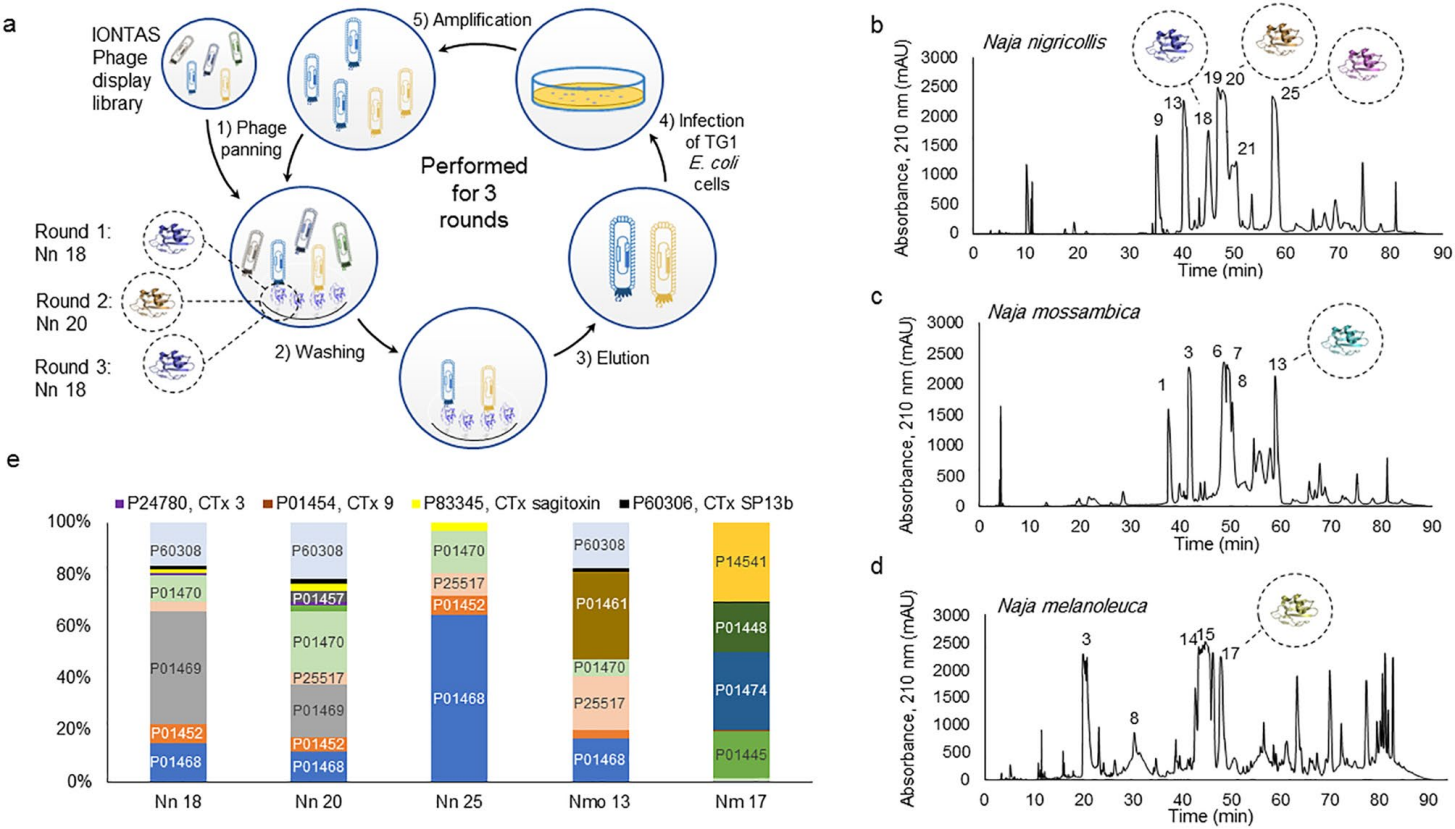
Overview of the cross-panning phage display strategy employed in this study and the venom fractions utilized. (**a**) In the cross-panning phage display strategy, the IONTAS phage display library was added to a vial, in which biotinylated cytotoxins were presented using streptavidin capture (**1**). While the phages displaying scFvs capable of binding to the target cytotoxin were bound, non-binding phages were washed away (**2**). The antigen-binding phages were eluted (using trypsin digestion) (**3**) and used to infect TG1 *E. coli* cells (**4**), upon which the phages were amplified (**5**) and used for the next panning round (**1**). It is important to note that the target antigen alternated between the panning rounds, in such a way that Nn 18 (blue) was used in round 1, Nn 20 (brown) used in round 2, and the first antigen (Nn 18) was used once again in round 3. (**b**–**d**) Cytotoxin-containing fractions that were utilized in this study have been highlighted on the corresponding chromatograms of the crude venoms of *Naja nigricollis* (Tanzania specimen), *N. melanoleuca* (Cameroon specimen), and *N. mossambica* (Tanzania specimen). (**e**) Accession numbers of the cytotoxins (or closest available homolog) found in each fraction using LC–MS/MS. The cytotoxin content of each fraction is normalized to 100%. Major proteins have been labelled on the graph, while trace proteins have been defined in the legend. The total cytotoxin content relative to total protein for each venom fraction was estimated to: 93% for Nn 18, 63% for Nn 20, 99.5% for Nn 25, 99% for Nmo 13, and 84.4% for Nm 17.

Accordingly, by developing and applying a phage display methodology relying on cross-panning against two different CTxs (Fig. 1a), we demonstrate the feasibility of discovering broadly-neutralizing human monoclonal antibodies capable of neutralizing CTxs from the venoms of three different cobras. We thereby also show that by strategically selecting which venom toxins to be used as antigens, we can select monoclonal antibodies that are able to neutralize not only the CTxs employed as antigens, but also homologous CTxs from venoms of different snake species.

## Material and methods

### Venom fractionation

Crude venoms from *N. nigricollis* (Tanzania), *N. mossambica* (Tanzania), and *N. melanoleuca* (Cameroon) were purchased in lyophilized form from Latoxan, France. Fractions 18, 20, and 25 from *N. nigricollis* (Nn 18, Nn 20, Nn 25), fraction 13 from *N. mossambica* (Nmo 13), and fraction 17 from *N. melanoleuca* (Nm 17) venoms were isolated by RP-HPLC (Agilent 1200 series) using a C18-column (Discovery BIO Wide Pore, 4.6 × 250 mm, 5 μm particle, 300 Å pore size, reversed phase) as described elsewhere^4^. Manually collected fractions were dried in a vacuum centrifuge, dissolved in phosphate buffered saline (PBS), pooled, and concentrations were estimated at 280 nm (NanoDrop One^C^ Spectrophotometer, Thermo Scientific).

### Proteomic-based characterization of the venom fractions

Individual vacuum dried fractions were re-suspended in 20 µL of 6 M guanidinium hydrochloride, containing 10 mM TCEP, 40 mM 2-Chloroacetamide and 50 mM HEPES pH 8.6. After adding 3 sample volumes of digestion buffer (10% Acetonitrile, 50 mM HEPES, pH8.5), fraction samples were digested with LysC endopeptidase (1:50; w:w) for 3 h at 37 ˚C. Then, after addition of the digestion buffer, samples were diluted 10 times and mixed with trypsin (1:100; w:w). Trypsinized samples were incubated O/N at 37 ˚C. Next, samples were diluted 2 times with 2% TFA to quench trypsin activity, and desalted on a StageTip containing Empore C18 with 12–16 µg peptide capacity, eluted in 40% Acetonitrile containing 0.1% TFA, dried in a vacuum centrifuge, and resuspended in LC–MS buffer (2% Acetonitrile, 1% TFA).

Mass spectrometry data was collected using a Q Exactive mass spectrometer (ThermoFisher Scientific, San Jose, CA) coupled to a Proxeon EASY-nLC 1200 liquid chromatography (LC) pump (ThermoFisher Scientific). Peptides were separated for 45 min on a 50 cm × 75 µm microcapillary PepMap RSLC C18 resin (2 µm, ThermoFisher Scientific), packed inside an EasySpray ES803A column. For analysis, 500 ng were loaded onto the analytical column. Full MS spectra were collected at a resolution of 70,000, with an AGC target of 3 × 10^6^or maximum injection time of 20 ms and a scan range of 300–1,750 m/z. The MS2 spectra were obtained at a resolution of 17,500, with an AGC target value of 1 × 10^6^ or maximum injection time of 60 ms, a normalised collision energy of 25 and an intensity threshold of 1.7 × 10^4^. Dynamic exclusion was set to 60 s, and ions with a charge state < 2 or unknown were excluded.

MS raw data files were searched against a custom, concatenated database, consisting of all available protein sequences in Uniprot for the *Naja* species combined with a toxin specific protein sequence database curated in-house. For standard database searching, the peptide fragmentation spectra (MS/MS) were analyzed by Proteome Discoverer 2.2. The MS/MS spectra were searched using the built-in Sequest HT algorithm and was configured to derive fully-tryptic peptides using default settings and label-free quantitation (LFQ) functionality.

### Biotinylation of the venom fractions

Biotin linked to *N*-hydroxysuccinimide (NHS) via a PEG_4_-linker (EZ-Link NHS-PEG_4_-Biotin, No-Weigh Format, Thermo Scientific, 21329) was added to the toxin containing solutions at a molar ratio of 1:1.5 (venom fraction:biotinylation reagent) and left at room temperature for 30 min. Buffer exchange columns (Vivacon 500, Sartorius, 3000 Da Molecular Weight Cut-Off) were used for purification of the biotinylated fractions following the manufacturer’s protocol. Concentrations of the biotinylated fractions were measured using the NanoDrop Spectrophotometer previously described.

### Phage display selection strategies

The IONTAS human single-chain variable fragment (scFv) antibody phage library containing a clonal diversity of 4 × 10^10^ was employed for phage display selections. Selection of scFvs was performed as described elsewhere^4,12^ with the important modification that a cross-panning strategy was employed, in which the antigen was alternated between the two selected CTxs over three rounds of panning: In the first round, the phage library was panned against Nn 18, in the second round against Nn 20 that included a change in capture vehicle from streptavidin to neutravidin and omitted deselection with Dynabeads, followed by a third round against Nn 18 antigen, as performed in round 1.

### Sub-cloning and direct ELISA

*Nco I* and *Not I* restriction endonucleases were used for sub-cloning of selected scFv genes from the phage vector into the pSANG10-3F vector for expression of soluble scFvs^13^ and transformed into *Escherichia coli* (*E. coli*) strain BL21(DE3) (New England Biolabs). A total number of 552 scFv-expressing clones that were obtained in the cross-panning experiments were selected and expressed in 96-well polypropylene microtiter plates. After replicating them in auto-induction media^13^, scFv-containing supernatants were tested for binding to biotinylated Nn 18 and Nn 20 venom fractions indirectly immobilized on streptavidin-coated MaxiSorp plates. Fraction 8 from a different snake species, *Dendroaspis polylepis,* was used as a negative control. A 20,000-fold dilution of Monoclonal Anti-FLAG M2-peroxidase covalently conjugated to horseradish peroxidase (Sigma, A8592) was used for detection of binding. A total number of 113 cross-binding scFv clones were identified, from which 46 were submitted for sequencing (Eurofins Genomics sequencing service) using the S10b primer (GGCTTTGTTAGCAGCCGGATCTCA). The third complementarity determining region of the heavy and light chains (CDRH3 and CDRL3, respectively) were analyzed to identify unique clones.

### scFv expression and purification

The TPL0027_01_F7 scFv clone was inoculated into 2xYT medium supplemented with 2% (w/v) glucose and 50 μg/mL kanamycin (2xYT-GK) and incubated O/N at 250 rpm, 30 °C. 500 mL auto-induction medium was inoculated using the O/N cultures and incubated O/N at 30 °C, 200 rpm. The cultured cells were centrifuged at 4,300 × *g* for 10 min, supernatant was discarded, and the pellet was re-suspended in 50 mL TES-buffer (30 mM Tris–HCl pH 8.0, 1 mM EDTA, 20% sucrose (w/ v)) containing 25 U/mL Benzonase Nuclease (Millipore, E1014-25KU), 2 μL/mL 25x cOmplete Protease Inhibitor Cocktail (Roche, 11836145001) and 1.5 kU/mL r-lysozyme (~ 70,000 U/mg, Sigma Aldrich, 62971-10G-F). After 20 min of incubation on ice, the cells were centrifuged at 4,300×*g* for 10 min, supernatant was decanted, and the cell pellet kept on ice. The cell pellet was re-suspended in 50 mL of 5 mM MgSO_4_ supplemented with similar amounts of Benzonase Nuclease and r-lysozyme as above, 40 μL/mL cOmplete Protease Inhibitor Cocktail, and incubated on ice for 20 min. After centrifugation at 4,300×*g* for 10 min, supernatant was pooled with the supernatant from the previous step and kept on ice. The pooled supernatants were centrifuged once again at 30,000×*g* for 30 min. Purification of His-tagged scFvs was performed using HisTrap FF 1 mL columns (GE Life Sciences, 17531901), which later were loaded on to an ÄKTAprime plus system (GE Healthcare) in order to elute the scFvs from the columns. Protein concentration was determined at 240 nm, assuming 1 absorbance unit is equivalent to 1 mg/mL of protein.

### Determination of half-maximal inhibitory concentration (IC_50_)

Immortalized human keratinocyte (N/TERT) cells were cultured in Dulbecco's modified Eagle's medium (DMEM: F12; Grand Island, NY, USA) supplemented with 10% (v/v) fetal bovine serum (FBS), 1% (v/v) penicillin–streptomycin (Sigma, St. Louis, MO, USA), and 1 × RMplus supplement. Cells were incubated O/N at 37 °C, 5% CO_2_, and 85% humidity. Approximately 4 × 10^3^ N/TERT cells were seeded in each well of 96-well polystyrene black opaque-plates (Thermo Fisher Scientific, Roskilde, DK)^14^. Then, the culture media was aspirated and 100 µL keratinocyte media containing a range of different concentrations of cytotoxins (Nn 18, Nn 20, Nn 25, Nm 17, and Nmo 13) was added. As controls, wells with either no cells or no toxin were used. Plates were incubated at 37 °C, 5% CO_2_, and 85% humidity for 24 h. To determine the percentage of viable cells, the CellTiter-Glo luminescent cell viability assay (Promega, Madison, WI, USA) was performed according to the manufacturer’s protocol. The IC_50_ values were calculated with GraphPad software (version 6; GraphPad Software, San Diego, CA), using the log(inhibitor) versus normalized response and a Hill equation.

### Cell cytotoxicity neutralization assay

A cell cytotoxicity neutralization assay was performed by subjecting approximately 4 × 10^3^ N/TERT cells to two times the IC_50_ of each venom fraction either in the absence or presence of different molar ratios of venom fraction:scFv (1:1, 1:2, and 1:3). Buffer and scFv-only controls were run in parallel and all samples were pre-incubated (30 min at 37 °C) prior to addition to the N/TERT cells. Experiments were performed in triplicates, and results were expressed as mean ± SD. One-way analysis of variance (ANOVA) followed by Tukey’s post hoc test was performed for each group (except for the Nn 18 group) and *p* < 0.05 was considered to be of statistical significance. For the Nn 18 group, a one-way ANOVA could not be carried out because the group only contained two samples, therefore an unpaired *t* test was performed with *p* < 0.05 considered to be of statistical significance. The Graphpad Prism Software was used for statistical analysis.

## Results

Venoms from *N. nigricollis*, *N. mossambica*, and *N. melanoleuca* were fractionated using RP-HPLC^7^ (Fig. 1b–d) and the following fractions were collected: Fraction 18, 20, and 25 from *N. nigricollis* (Nn 18, Nn 20, Nn 25), fraction 13 from *N. mossambica* (Nmo 13), and fraction 17 from *N. melanoleuca* (Nm 17). Using LC–MS/MS and analyzing raw data against the genus (*Naja*) entries in the Uniprot database, a total number of 17 distinct CTxs were identified in the five fractions of interest (Fig. 1e).

Fractions Nn 18 and Nn 20 were biotinylated and subjected to phage display selection using the IONTAS human scFv library; an updated version of the library developed by Schofield *et al*.^12^ created from a naïve human B-cell repertoire with a diversity of 4 × 10^10^ M13 bacteriophages displaying human scFv fragments fused to the pIII protein. Phage display selection was performed as previously described^4^, however, with the important detail that the library was cross-panned against two antigens in an alternating fashion (Nn 18 in round 1, Nn 20 in round 2, and Nn 18 in round 3) (Fig. 1a). Upon confirmation of successful amplification of binders, scFv genes were isolated from both the second and third panning rounds, sub-cloned into the pSANG10-3F vector, transformed into BL21(DE3) *E. coli* cells, and expressed as soluble scFvs, which were used to assess binding characteristics in direct ELISA^4^.

**Figure 2:**
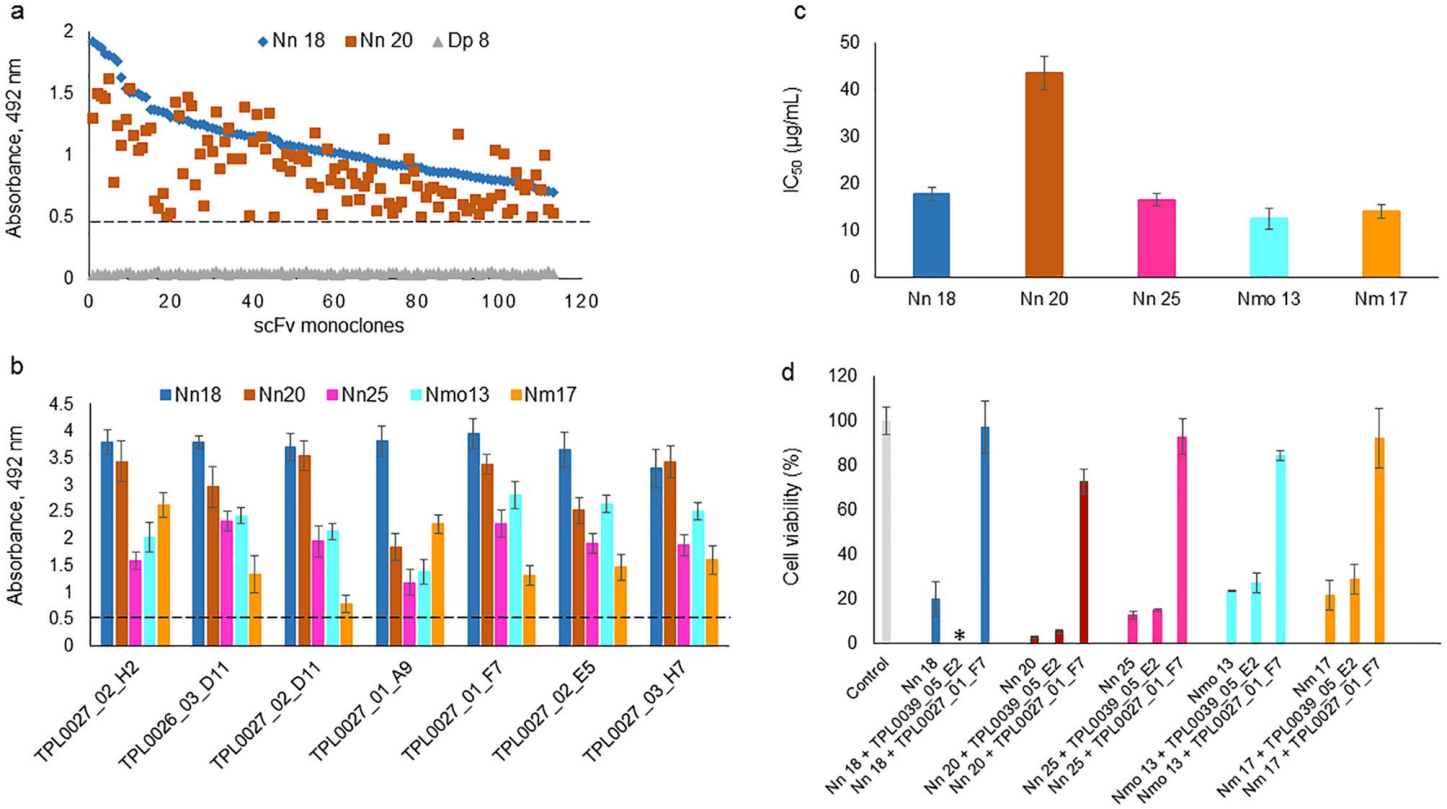
Binding and neutralizing capability of the scFv clones discovered using phage display and a cross-panning strategy against CTxs from African cobras. (**a**) In direct ELISA, 113 cross-binding scFv clones recognized both Nn 18 and Nn 20 venom fractions. Fraction 8 from *Dendroaspis polylepis* venom (Dp 8, Dendrotoxin 1) was used as a negative control. (**b**) Seven of the unique cross-binding scFv clones bound five different CTxs-containing fractions from three different cobra species (Nn 18, Nn 20, Nn 25, Nmo 13, and Nm 17). Bars represent mean ± SD of triplicate wells. (**c**) Different amounts of venom fractions (2–80 µg/mL) were added to N/TERT cells. After 24 h of incubation at 37 °C, cell viability was determined by quantifying the ATP release into the culture media, and mean cell survival percentage was calculated from triplicate wells. The half-maximal inhibitory concentration (IC_50_) was determined by fitting the mean cell survival percentage of inhibition-concentration data into a Hill equation and has been represented as values ± standard error. (**d**) Neutralization of the cytolytic effects of the venom fractions on human N/TERT cells by the discovered broadly-neutralizing TPL0027_01_F7 scFv antibody. 2 IC_50_ of the venom fractions were pre-incubated with TPL0027_01_F7. The scFv antibody TPL0039_05_E2, targeting the unrelated Myotoxin II from *Bothrops asper* venom, was used as a negative control. Experiments were performed in triplicates, and results are expressed as mean ± SD. One-way analysis of variance (ANOVA) followed by Tukey’s post hoc test was performed for each group, except the Nn 18 group, and *p* < 0.05 was considered to be of statistical significance. For the Nn 18 group, a one-way ANOVA could not be carried out because the group only contained two samples, therefore, an unpaired *t* test was carried out with *p* < 0.05 considered to be of statistical significance. For each of the five fractions, there was a statistically significant difference between “fraction + TPL0027_01_F7” and “fraction”. For the four groups containing the “fraction + TPL0035_09_E2”, there was a statistically significant difference between this and “fraction + TPL0027_01_F7”. However, differences between “fraction” and “fraction + TPL0035_09_E2” were not statistically significant for any of the four groups. *Insufficient amounts, this fraction was not available for testing.

Among the total number of 552 scFv clones that were screened, 113 cross-binding scFv clones recognized both fractions (Fig. 2a). The genes for the top 46 cross-binding scFv clones were DNA sequenced, yielding 24 cross-binding scFvs with unique CDRH3 and/or CDRL3 amino acid sequences. The unique scFv clones were further tested for cross-binding against a broader range of CTxs from the same and other spitting and non-spitting cobra species. It was speculated that the scFvs raised against Nn 18 and Nn 20 may recognize structurally similar antigens that are found in the fractions Nn 25, Nmo 13, and Nm 17 (Fig. 1e). Notably, seven of the unique cross-binding scFv clones were demonstrated to possess cross-binding ability against all five different fractions in the direct ELISAs (Fig. 2b). To rank the binding capacity of the seven unique scFvs, an expression-normalized direct ELISA was performed, in which TPL0027_01_F7 was identified as the most promising cross-binding scFv clone.

The TPL0027_01_F7 scFv clone was expressed, purified, and used to assess whether (cross-)binding would translate into (cross-)neutralization^15^. Given that snake CTxs mediate cytolytic effects and cause degradation and destruction of skin and connective tissue in vivo^9^, an in vitro skin-mimicking assay was used to serve as a surrogate for in vivo experimentation aiming to reduce animal suffering^3^. Hence, an immortalized human keratinocyte cell line (N/TERT cells) was subjected to different concentrations of fractions, either in the presence or absence of the TPL0027_01_F7 antibody fragment. Additionally, a non-specific scFv clone (TPL0039_05_E2) selected against Myotoxin II, a phospholipase A_2_ from *Bothrops asper* venom, was included as a negative control antibody. The toxicity of each CTx represented by the half-maximal inhibitory concentration (IC_50_) and the neutralization capacity of the scFvs were evaluated in a cell viability assay.

It was apparent that four of the venom fractions (Nn 18, Nn 25, Nmo 13, and Nm 17) exerted a comparable potent cytolytic effect on the N/TERT cells, demonstrated by low IC_50_ values ranging between 12 and 18 µg/mL, while one of them (Nn 20) was less potent and required almost three times the amount (43 µg/mL) to cause a similar effect (Fig. 2c). The ability of TPL0027_01_F7 to inhibit the cytolytic effect of the five fractions containing more than a dozen different CTxs is shown in Fig. 2d. To achieve 100% cytolytic effect of the venom fractions, 2 IC_50_s of each venom fraction were used. However, except for Nn 20, the venom fractions did not demonstrate a 100% cytolytic activity and around 20% of the cells remained alive. Yet, it was remarkable that at a molar ratio of 1:3 (preincubated venom fraction to TPL0027_01_F7 antibody), more than 90% of cells remained viable in the presence of Nn 18, Nn 25, and Nm 17, whereas 83% cell viability was achieved for Nmo 13 and 70% for Nn 20. In comparison, the non-specific scFv, TPL0039_05_E2, that was previously developed in our group, demonstrated no neutralization effect when used as negative control. Indeed, in the cells treated with a pre-incubated mixture of the venom fractions and TPL0039_05_E2 scFv (1:3 molar ratio), cell viability was similar to the level observed for cells treated with the venom fractions alone, demonstrating that the neutralization capacity of TPL0027_01_F7 did indeed derive from specific toxin binding.

## Discussion

Monoclonal antibodies are often exploited as therapeutic agents due to their high specificity and affinity for target antigens. However, high mono-specificity is not necessarily favourable when multiple antigens need to be neutralized, as in the case of animal venoms that comprise multitudes of similar and dissimilar toxins. In such cases, it is beneficial, if not essential, to use broadly-neutralizing monoclonal antibodies that are able to target multiple (similar) antigens, as it is critical to keep the number of monoclonal antibodies in a therapeutic product low to allow for feasible biomanufacture^16^. In this relation, development of human monoclonal antibodies that can broadly neutralize different venom toxins is key in the field of recombinant antivenoms, since this therapeutic property is essential for cost-competitive manufacture of novel antivenom products^6,17^. It is, however, also important to assess what antibody format, i.e. monovalent fragment antigen binding (Fab) or bivalent immunoglobulin G (IgG), might present the most optimal therapeutic properties in terms of pharmacokinetics (i.e. half-life and penetration rate into deep tissue) and pharmacodynamics (i.e. improved neutralization via avidity effects), as these features may also impact efficacy and cost of manufacture^18^.

The discovery and development of broadly-neutralizing monoclonal antibodies is a complex endeavor, which in the field of animal envenoming therapy has so far only been achieved with time and labor-intensive trial and error methods, based on directed evolution and site-directed mutagenesis approaches, employing *Centroides* scorpion neurotoxins as target antigens^19^ or by plain serendipity using *Bothrops* snake venom as the target^20^. In contrast, we here report a new methodology, based on a phage display and cross-panning strategy, which allows for systematic and facile discovery of broadly-neutralizing toxin-targeting antibodies in a single experiment. This methodology may find its utility in the development of broadly-neutralizing (polyvalent) recombinant antivenoms with wide species coverage^18^. However, it has not escaped our attention that this methodology may also be applicable for the development of antibody-based therapeutics against a range of other indications involving multiple toxins or elusive pathogens, such as infectious diseases with toxin-excreting bacteria, viruses prone to hyper-mutation, or parasites with a huge capacity for antigen variation.
